# Miniature chicken ileal explant culture to investigate the inflammatory response induced by pathogen-associated molecular patterns

**DOI:** 10.3389/fvets.2025.1484333

**Published:** 2025-03-18

**Authors:** Gábor Mátis, Csilla Sebők, Dávid G. Horváth, Rege Anna Márton, Máté Mackei, Júlia Vörösházi, Ágnes Kemény, Zsuzsanna Neogrády, Ilona Varga, Patrik Tráj

**Affiliations:** ^1^Division of Biochemistry, Department of Physiology and Biochemistry, University of Veterinary Medicine, Budapest, Hungary; ^2^National Laboratory of Infectious Animal Diseases, Antimicrobial Resistance, Veterinary Public Health and Food Chain Safety, University of Veterinary Medicine, Budapest, Hungary; ^3^Department of Pathology, University of Veterinary Medicine, Budapest, Hungary

**Keywords:** immunomodulation, explant, poultry, cytokines, flagellin, lipoteichoic acid, poly I:C

## Abstract

Gastrointestinal inflammation leads to maldigestion and systemic diseases in poultry. To tackle the problem of the industry and to search for therapeutic candidates *in vitro* models are inevitable. Both immersion and air-liquid interface explant models are available, although there is limited information on the size-dependent applicability and response to different pathogen-associated molecular patterns (PAMPs) in the case of these model systems. The study aimed to compare the morphology and viability of miniature chicken gut explant cultures obtained with a biopsy punch to examine the size-dependent change over time. To verify the applicability of the model, pathogen-associated molecular patterns (PAMPs): flagellin, lipoteichoic acid (LTA) and polyinosinic polycytidylic acid (poly I:C) were applied to induce inflammation. The 2 mm diameter explants showed a decrease in metabolic activity measured by CCK-8 assay after 12 h and a significantly higher extracellular lactate dehydrogenase activity indicating cellular damage compared to the 1 mm explants, supported by histological differences after 24 h of culturing. After 12 h of incubation, the 1.5 mm explants retained columnar epithelial lining with moderate damage of the lamina propria (H&E and pan-cytokeratin staining). Exposure to 100 μg/mL poly I:C reduced the metabolic activity of the 1.5 mm explants. LTA and poly I:C increased IFN-*γ* concentration at both applied doses and IFN-*α* concentration was elevated by 50 μg/mL poly I:C treatment. Flagellin administration raised IL-2, IL-6, and RANTES levels, while higher LTA and poly I:C concentrations increased the IFN-*γ*/IL-10 ratio. According to the observations, the viability and integrity of the explants decreases with their size. After 12 h, the 1.5 mm diameter miniature chicken ileal explant stimulated with PAMPs can be an appropriate model to mimic diseases involving tissue damage and inflammation.

## 1 Introduction

The gastrointestinal tract represents the body’s largest surface exposed to the external environment (1). The gut tolerates feed and commensal antigens but acts when a pathogen is detected. However, the impairment of the enterocyte layer enables the molecules of extraneous origin to come into contact with the receptors of the innate immune cells. The first alarm goes off as the pathogen-associated molecular pattern (PAMP) binds to its corresponding Toll-like receptor (TLR). Subsequently, an intracellular signal transduction begins, leading to immense proinflammatory cytokine production. The alert with the cytokines spreads further and calls forth a proinflammatory response on the level of dendritic cells and the abundantly present intraepithelial lymphocytes (2). *In vitro* models are inevitable to investigate these intricate mechanisms. *In vitro* cultures stimulated with PAMPs serve as models to challenge nutraceuticals, probiotics, and drugs with potential anti-inflammatory effects (3).

In animals, one of the primary causes of dysbiosis and disruption of intestinal immunological homeostasis is an enteric infection caused by bacteria, viruses, or parasites (4). The intestinal epithelium expresses most TLR types on the luminal surface; however, the majority of these TLRs are not intrinsically responsive to PAMPs. Nevertheless, proinflammatory cytokines such as interferon-*γ* (IFN-γ) and tumor necrosis factor-alpha (TNF-*α*) could increase their abundance and responsiveness (5). The intestinal mucosa is a functional aggregation of neighborhoods and niches of roughly 20 types of immune and epithelial cells (6). Growing evidence suggests intestinal mucosal inflammation and barrier function are defined and regulated by the interaction of distinct cell types such as enteric glial cells, intraepithelial lymphocytes, and some epithelial cell types with different commitments and functions. Therefore, the structure determines the response (7, 8).

Gut explants are cultures made of gut or gut mucosa fragments and maintained *ex vivo* to recapitulate the most important characteristics of the organ in the live animal. Compared to gut epithelial cell cultures, the fundamental advantage of this model is the polarized and layered structure with vital cell–cell interactions. Intestinal explants could reproduce the tissue-specific cytokine production of the gut (7, 9, 10). Explant culturing requires an oxygen-rich environment and liquid-tissue-gas interface since anoxia is the primary cause of cell necrosis in explant cultures (11). It makes the *ex vivo* explant method technically complicated and challenging to conform with the general requirements of cell culturing, e.g., a high number of replicates and treatment groups. One potential approach to overcome this technical limitation is to decrease the tissue sample size (12, 13). The smaller the explant sample and the younger the donor is, the higher the apparent resistance of the explant to anoxia (7). Moreover, some explant methods in the existing literature on the topic report the application of surgically removed biopsy samples or cut slices with approximate size. Most protocols available do not suggest how to gain explants with consistent size and shape.

The present study investigates if biopsy punches under 2 mm diameter genuinely used for the sectioning of gut explants could be applied to obtain tissue samples with identical size and similar morphology. To assess the hypothetically size-dependent viability and morphology of these *ex vivo* cultures over time and to select the ideal diameter and incubation time, the morphology of 1 and 2 mm explants were compared and their viability was followed for 36 h in study 1. The morphology of a potentially applicable explant was further evaluated in study 2 with H&E stain and pan-cytokeratin immunohistochemistry before and after 12 h culturing. Afterward, the ileal explant with the chosen diameter was stimulated with flagellin, lipoteichoic acid (LTA) and polyinosinic polycytidylic acid (poly I:C) to validate its inflammatory cytokine production and assess the change in its viability caused by exposure to different PAMPs.

## 2 Materials and methods

### 2.1 Experimental animals

For the experiments, three 21-day-old male Ross-308 broiler chickens were sacrificed in line with the chicken ileal explant model of Zhang et al. and the chicken hepatic culture models of the authors (3, 10, 14). Each study (study 1, 2 and 3) was executed after the extermination of one individual, considering the homogeneity of the cultures and 3R principles. The animals were handled following the animal welfare legislation of the European Union, institutional policies, and the Local Animal Welfare Committee of the University of Veterinary Medicine Budapest. The chickens were kept and fed in conformity with Ross Technology. The experiment was approved by the Government Office of Zala County, Food Chain Safety, Plant Protection and Soil Conservation Directorate, Zalaegerszeg, Hungary (license number GK-419/2020; approval date: May 11, 2020).

### 2.2 Materials

The used Penicillin–Streptomycin (Pen-Strep) is a product of Gibco (Waltham, MA, USA), and HCMTM SingleQuotsTM Kit (Catalog No. CC-4182) is a product of Lonza-Biocenter (Szeged, Hungary). The latter contains ascorbic acid, bovine serum albumin, hydrocortisone, transferrin, human Epidermal Growth Factor (hEGF), insulin, gentamicin and amphotericin-B. All other chemicals applied were obtained from Merck KGaA (Darmstadt, Germany). Reusable biopsy punches with plungers were purchased from MDE GmbH (Heidelberg, Germany).

### 2.3 Tissue sample isolation and excision of the explants

Before the aseptic opening of the coelomic cavity, the animal was decapitated under CO_2_ anesthesia and restrained in a dorsal position. Then 15 cm long ileal segment was excised 10 cm distally from the Meckel’s diverticulum. The adipose tissue was removed from this intestinal section by hand. The outer side was washed with phosphate-buffered saline (PBS) + Pen-Strep solution (1%) and the removed section was flushed from both directions using a stainless-steel feeding needle according to Udden et al. (15). The intestinal section was cut longitudinally on the mesenteric side and washed three consecutive times with PBS + Pen-Strep solution. This step might be repeated until no physical contaminants are visible. After that, it was cut lengthwise into four pieces, which were placed in ice-cold fresh PBS + Pen-Strep solution and were further handled on ice. For culturing 24- and 96-well plates (Greiner Bio-One Hungary Kft., Mosonmagyaróvár, Hungary) coated with collagen type I (10 g/cm2) were applied. Before excising the explants, the wells of the required culture plates were filled with culture medium (24-well plates - 400 μL medium, 96-well plates - 100 μL medium). The medium used throughout the experiment was Dulbecco’s Minimal Essential Medium-F12 supplemented with FBS (fetal bovine serum) 2.5%, glutamine 1%, Pen-Strep 1% and one package of HCMTM SingleQuotsTM Kit. The explants were cultured at 37°C under 5% CO_2_. The excision of the explants was carried out as follows:

1. One intestinal segment was placed, mucosa side up, on a sterile, chilled glass, and then the segment was gently and evenly pressed against the glass tile using glass slides.
2. The surface of the intestinal samples was rinsed frequently with ice-cold PBS + Pen-Strep solution using a syringe during the excision of the explants to keep the tissue moist.
3. Explant removal was performed with a biopsy punch perpendicular to the glass plate, moving the punch in a circular motion.
4. The excised specimens were transferred directly into the prepared wells with the medium by the plunger of the instrument.
5. Peyer’s patches were avoided, and explant replicates were sectioned in a row for each experimental group to minimize the potential difference between groups.

### 2.4 Culturing and treatment of the explants

#### 2.4.1 Study 1

The viability, cell membrane damage and histology of 1 and 2 mm diameter explant cultures were assessed. The cultures were obtained according to section 2.3. Six 1 and 2 mm diameter explants were fixed instantly with formaldehyde solution on biopsy sponges at room temperature for further evaluation (H&E stain). The remaining explants were directly deposited into the cell culture plate wells. 1 mm explants were cultured on 96-well plates in 100 μL and the 2 mm explants were placed in the wells of 24-well plates in 400 μL media proportionately to the volume of the explant to make the biological response of 1 and 2 mm explants comparable. The shape of the 1 and 2 mm explants were cylinder-like with the same height, therefore the volume was calculated and compared according to the formula of the volume of a cylinder. Volumes were kept at a low minimum to ease the diffusion of gasses to the explants. The wells were allocated to have *n* = 6 separate explants for 24 h histology (H&E stain and pan-cytokeratin immunohistochemistry), metabolic activity and lactate dehydrogenase measurements (H&E stain and pan-cytokeratin immunohistochemistry) from both, the 1-and the 2 mm diameter explants. The media of the explants was changed every 12 h, and the removed medium samples were stored for LDH measurement in the case of separate LDH groups of explants. A CCK-8 assay was performed with concomitant medium changes before and after a measurement on the separate CCK-8 groups. In case of the 24 h histology groups a simple change of the medium has been carried out. Most of the 2 mm explants for the CCK-8 and LDH tests were macroscopically disrupted after approximately 48 h of culturing, therefore the measurements were terminated after 36 h. 1 mm samples were maintained longer with regular medium changes every other day. After 9 days, the study was concluded, and the 1 mm explants were fixed on the plate wells and stained according to Giemsa.

#### 2.4.2 Study 2

Based on the results of Study 1 and for practical reasons (The repetitive change of media can be technically difficult in case of 1 mm explants.), the smallest obtainable and manageable, 1.5 mm diameter explants were excised. Five explants were placed in 200 μL of medium on 96 well plates. The medium was changed 1 h after seeding. The explants were left untreated for 12 h, fixed with formalin on biopsy sponges, and stored for H&E staining and pan-cytokeratin immunohistochemistry.

#### 2.4.3 Study 3

Based on the results of Study 1 and Study 2 1.5 mm diameter explants were obtained. The samples (*n* = 5/group) were placed in 200 μL of medium on 96 well plates. The medium was changed 1 h after seeding for the treatment media supplemented with the following chimicals in six separate groups: 10 or 50 μg/mL LTA from *Staphylococcus aureus* or 100 and 250 ng/mL *Salmonella Typhimurium*-derived flagellin, or with 50 and 100 μg/mL poly I:C for 12 h, respectively. The concentrations were selected based on preliminary results and previous studies on chicken cell cultures (3). Poly I:C was heated at 50°C for 3 min and then cooled down before adding it to cell culture media to achieve the re-annealing. The culture media were collected after 12 h and stored at −80°C until further analyses. The viability of the cultures was tested at this time point using CCK-8 method.

### 2.5 Measurements and histology

#### 2.5.1 Metabolic activity

Metabolic activity of explants was measured by CCK-8 assay (Cell counting Kit-8, Dojindo Molecular Technologies, Rockville, MD, USA) after 1, 12, 24 and 36 h for Study 1 and after 12 h for Study 3. CCK-8 reagent working solution was diluted in a ratio of 1:10 with fresh medium and it was pipetted on the explants. After 1 hour of incubation under culturing conditions, 100 μL of culture media sample was transferred to an empty 96-well plate to measure the absorbance according to the manufacturer’s instructions at 340 nm with a Multiskan GO 3.2 reader (Thermo Fisher Scientific, Waltham, MA, USA). As the amount of formazan produced is proportional to the amount of NAD(P)H + H^+^ produced, the color intensity can be used to measure the intensity of the cellular catabolic processes, shortly the metabolic activity.

#### 2.5.2 Extracellular lactate dehydrogenase (LDH) activity

The LDH activity is commonly applied as the tissue viability/breakdown parameter of *ex vivo* cultures (10, 16, 17). The high LDH activity in the medium indicates that the cell membranes are damaged, and the intracellular enzyme is released into the medium. The extracellular LDH enzyme activity indicating membrane damage was determined by a kinetic photometric assay from samples of culture medium obtained after 1, 12, 24 and 36 h for Study 1 and after 12 h for Study 3. A Lactate Dehydrogenase Activity Assay Kit (Merck KGaA, Darmstadt, Germany) was utilized to measure the parameter. The enzyme reduces NAD+ to NADH+H+, which is detectable at 450 nm with a photometric reader. 50 μL of culture medium samples diluted with LDH Assay Buffer were added to 96-well microplates and mixed with 50 μL of freshly prepared Master Reaction Mix. After 2 minutes of incubation at 37°C, the absorbance was measured for the first time at 450 nm using a Multiscan GO 3.2 reader (Thermo Fisher Scientific Inc., Waltham, MA, United States). Every 5 minutes, measurements were taken until the absorbance of the most active sample exceeded the value of the highest standard.

#### 2.5.3 Interleukin-8 (IL-8) concentration

According to the manufacturer’s instructions, IL-8 concentrations were determined from medium samples of Study 3 using a chicken-specific sandwich ELISA kit (MyBioSource, San Diego, CA, USA). Measurements were performed with a Multiskan GO 3.2. Values were normalized in GraphPad Prism and displayed as relative percentages. The smallest mean in the data set was defined as 0% and the largest as 100%.

#### 2.5.4 IFN-*α*, IFN-*γ*, IL-2, IL-6, IL-10, and RANTES concentration

According to the manufacturer’s recommendations, the Milliplex Chicken Cytokine/Chemokine Panel (Cat.Nr.: GCYT1-16 K, Merck KGaA, Darmstadt, Germany) medium was used to assess the protein concentrations of IFN-*α*, IFN-*γ*, IL-2, IL-6, IL-10 and RANTES in the medium samples of study 3. Blinded analysis was performed on a Luminex MagPix instrument. All samples in two technical replicates were included. Data collection employed Luminex xPonent 4.2 software. Five-parameter logistic regression curves were produced as standard curves for each analyte using the median fluorescence intensity values of the beads applying Millipore Belysa 1.1 software (Merck Millipore, Darmstadt, Germany). Values were normalized in GraphPad Prism and displayed as relative percentages. The smallest mean in each data set was defined as 0% and the largest as 100%.

#### 2.5.5 H&E and pan-cytokeratin immunohistochemistry

The explants from Study 1 directly after the sampling and after 24 h of culturing and in case of Study 2 after 12 h of culturing were fixed on foam biopsy pads. After 24 h of fixation at room temperature in formaldehyde solution, samples were trimmed and dehydrated with a series of ethanol and xylene in an automatic tissue processor. The dehydrated tissue samples were embedded in paraffin blocks with the orientation to allow the largest possible surface area to be cut. Thus 3-4 μm thin sections were made manually and mounted onto Superfrost+ adhesion slides (Thermo Fisher Scientific, Waltham, USA). The unstained sections were deparaffinized and rehydrated in xylene and alcohol, respectively. Routine H&E staining was performed using an automatic staining instrument. The H&E slides were examined to evaluate and compare the explants focusing on three criteria of Kolf-Clauw et al. investigated on a porcine jejunal explant culture, precisely the lesion of the tissue, the number of crypts and the enterocyte morphology (18).

To compare 1 and 2 mm samples from Study 1 after 24 h of culturing and to assess the morphology of the samples from Study 2 after 12 h of culturing, pan-cytokeratin immunohistochemistry was applied to evaluate the integrity of intestinal epithelium via the assessment of cytokeratin intermediate filaments. Sections were deparaffinized in xylene (2 × 15 min) and rehydrated in 96% and absolute ethanol for 5–5 min. It was followed by a 2x rinse in distilled water for a few minutes. Antigen retrieval was performed in EnVision FLEX target retrieval solution, High pH 9 (50x) in a microwave (800 Watts for 5 min, then 180 Watts for 10 min), then the sections were flushed with PBS 2 times. EnVision FLEX Peroxidase-Blocking reagent was added for 5 min, followed by a PBS rinse 3x (EnVision Flex Wash Buffer 20x). The primary antibody (Monoclonal Mouse Anti-Human Cytokeratin, clones AE1/AE3, Agilent Technologies) was added and incubated at a 1:200 dilution in a wet chamber for 30 min at room temperature. The slides were rinsed 2x in PBS before and after the 20-min incubation of the secondary antibody (Dako, EnVision Flex HRP). The staining was displayed with DAB Chromogen (Dako Envision Flex DAB + Chromogen 1 drop +1 mL Envision Flex Substrate Buffer) for 1–3 min and thereafter the sections were rinsed 2x in PBS. The slides were counterstained with hematoxylin according to GILL II for 10–15 s. Bluing was performed in PBS for 5 min. The slides were covered with coverslips after dehydration in ethanol and xylene (3-3 min in 96% and absolute ethanol, and 2 × 3 min in xylene).

### 2.6 Statistics

Statistical analysis was performed using R core Team software version 4.0.4 (19). Results were plotted as mean and standard error of the mean (SEM) on bar graphs using GraphPad Prism software. The distribution of the data was examined using a Q-Q plot. In Study 1 paired *T*-test was applied to compare the metabolic activity of the same explant group at different time points. An unpaired *T*-test was used to compare the metabolic activity and the LDH activity of 1 and 2 mm explants. In Study 3 group means were compared with those of the untreated control group, using Wilcoxon signed-rank test. The difference between means was considered significant if the *p*-value was less than 0.05.

## 3 Results

### 3.1 Study 1

**Figure 1:**
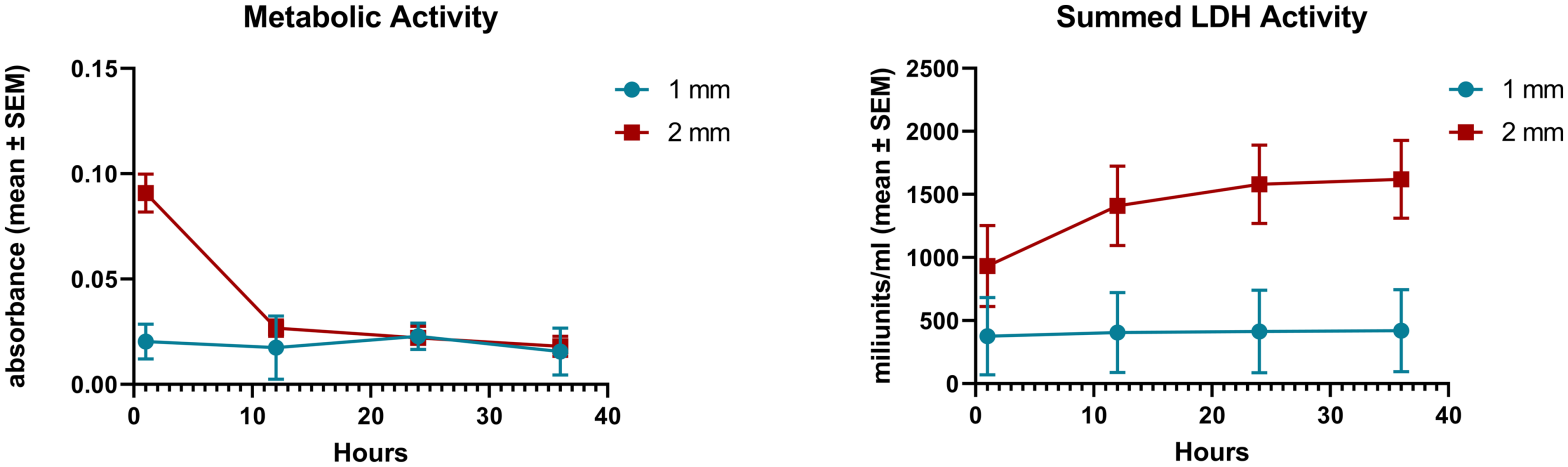
Cellular metabolic activity measured by CCK-8 assay and total lactate dehydrogenase (LDH) activity measured by an enzyme kinetic photometric assay of 1 and 2 mm explants. Mean (*n* = 5/group) ± SEM.

The cellular metabolic and total LDH activities of the explants were measured in the first 36 h of culturing. The initial metabolic activity of the 2 mm explants was remarkably higher than that of the 1 mm samples (*p* = 0.002), but the value of the 2 mm explants decreased significantly by 12 h (*p* = 0.001), hence there was no significant difference between the 1 and 2 mm explants from the 12 h time point on Figure 1. Concerning the total LDH activity of the medium samples the levels of the 2 mm explants from 12 h time point on were found significantly higher than that of the 1 mm samples (12 h *p* = 0.015, 24 h *p* = 0.006, 36 h *p* = 0.005) (Figure 1).

**Figure 2:**
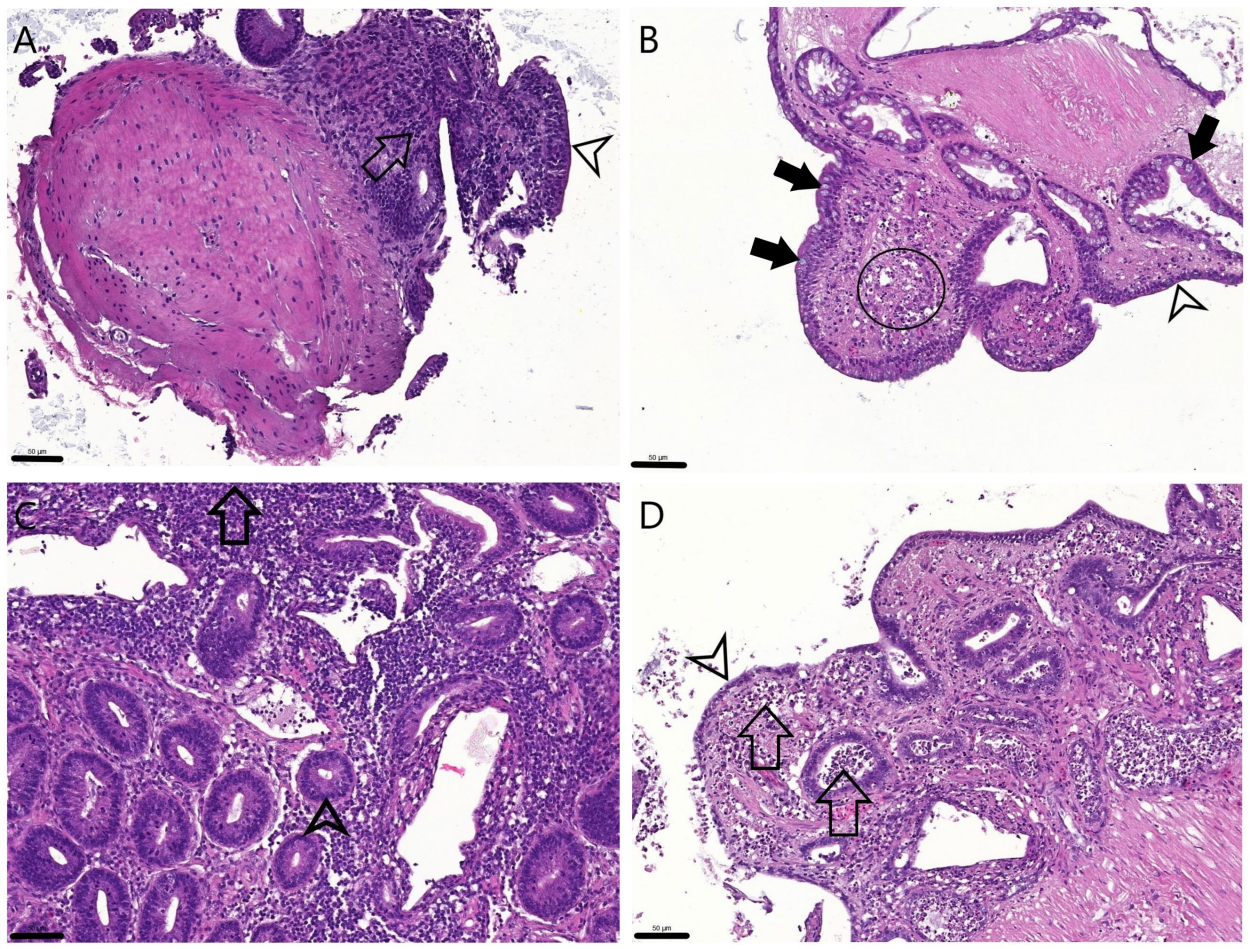
H&E stained sections of the explants. 0 h 1 mm **(A)** and 2 mm **(C)**: The physiologic columnar epithelial morphology (arrowheads) is visible on the surface and within the crypts, and the proprial mononuclear infiltrate is observable (arrows). 24 h 1 mm **(B)**: The cuboidal surface enterocytes (arrowhead) and Goblet cells (full arrows) are visible both inside the crypts and on the surface while in the lamina propria apoptotic cell debris appears (circle). 24 h 2 mm **(D)**: The surface enterocytes are flattened or detached (arrowhead), cell debris is apparent in the lamina propria and also within the crypts (arrows). Bar line = 50 μm.

Histology showed that the ileal epithelial cells lost their typical columnar morphology. After 24 h the epithelium of 1 mm samples was rather cuboidal while that of the 2 mm explants was more flattened. Moreover, necrotic and/or apoptotic cell debris was observed in the crypts and the lamina propria mainly of 2 mm samples after 24 h of culturing (Figure 2).

**Figure 3:**
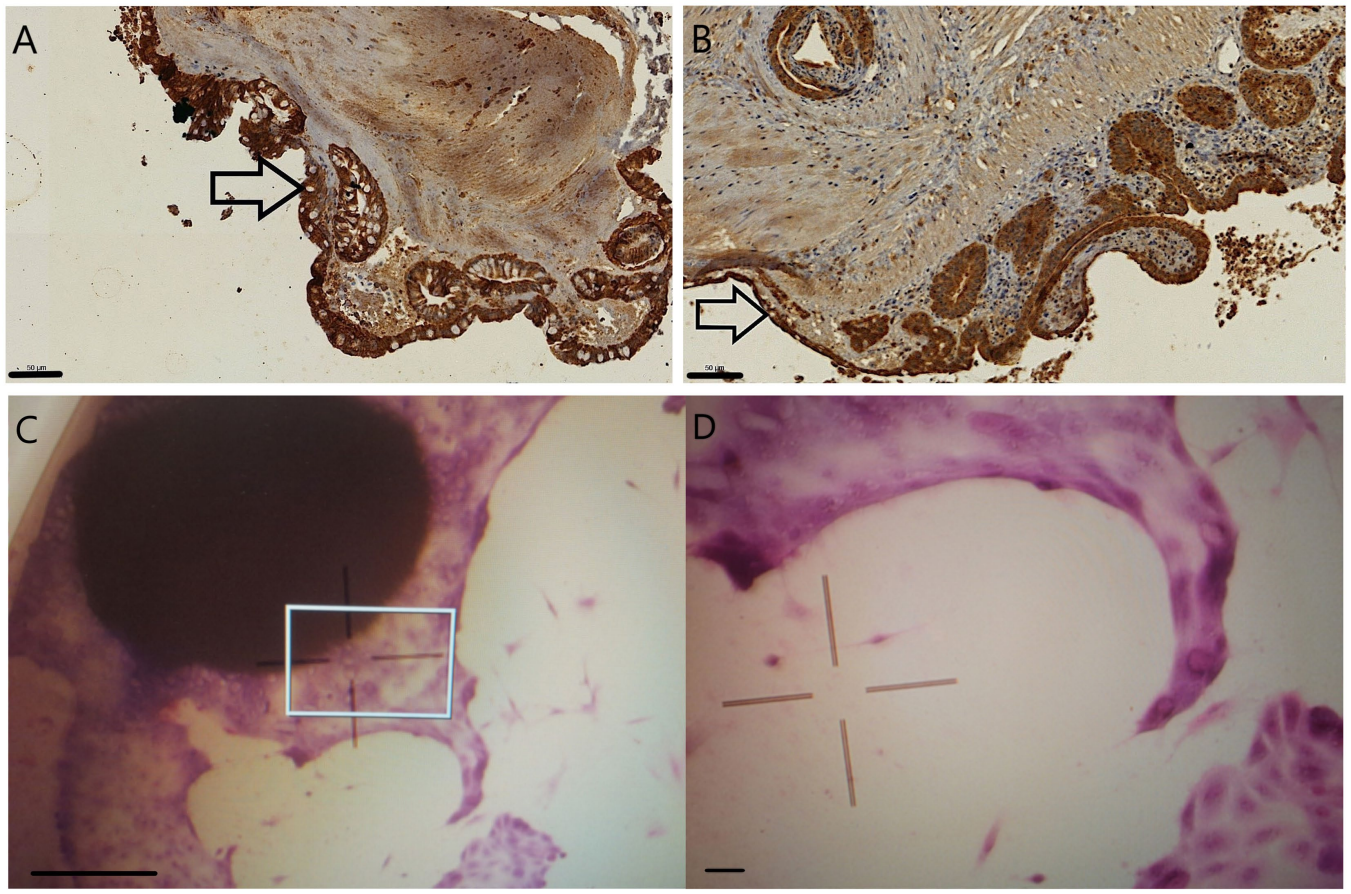
Pan-cytokeratin stained 24 h sections of 1 mm **(A)** and 2 mm **(B)** explants. The epithelial cells are marked in dark brown, and Goblet cells, with their unstained intracytoplasmic vacuoles, are primarily seen in 1 mm explants (arrows). Giemsa staining of the 1 mm explants after 9 days of culturing visualized with inverse microscope **(C,D)**: epithelial cells budding from the original explant tissue. The indicated area of picture **C** (square) was magnified to gain picture **D**. Bar line = 50 μm **(A,B,D)**, 500 μm **(C)**.

Pan-cytokeratin staining of explants after 24 h incubation highlighted presumably intact enterocytes and Goblet cells in 1 mm explants. The Goblet cell counts apparently decreased in the 2 mm samples. While tissue debris was more evident in the lamina propria of 2 mm specimens, intact inflammatory cells were also visible at this location in the 1 mm explants (Figures 3A,B).

The disruption of the 2 mm explants was evident after 48 h of culturing as they scattered into smaller pieces and cell debris, making their further manipulation and culturing impossible. After 9 days of culturing 1 mm, explants were macroscopically intact with epithelial cells budding out from their surface visible after fixation and Giemsa staining with inverse microscopy (Figures 3C,D).

### 3.2 Study 2

**Figure 4:**
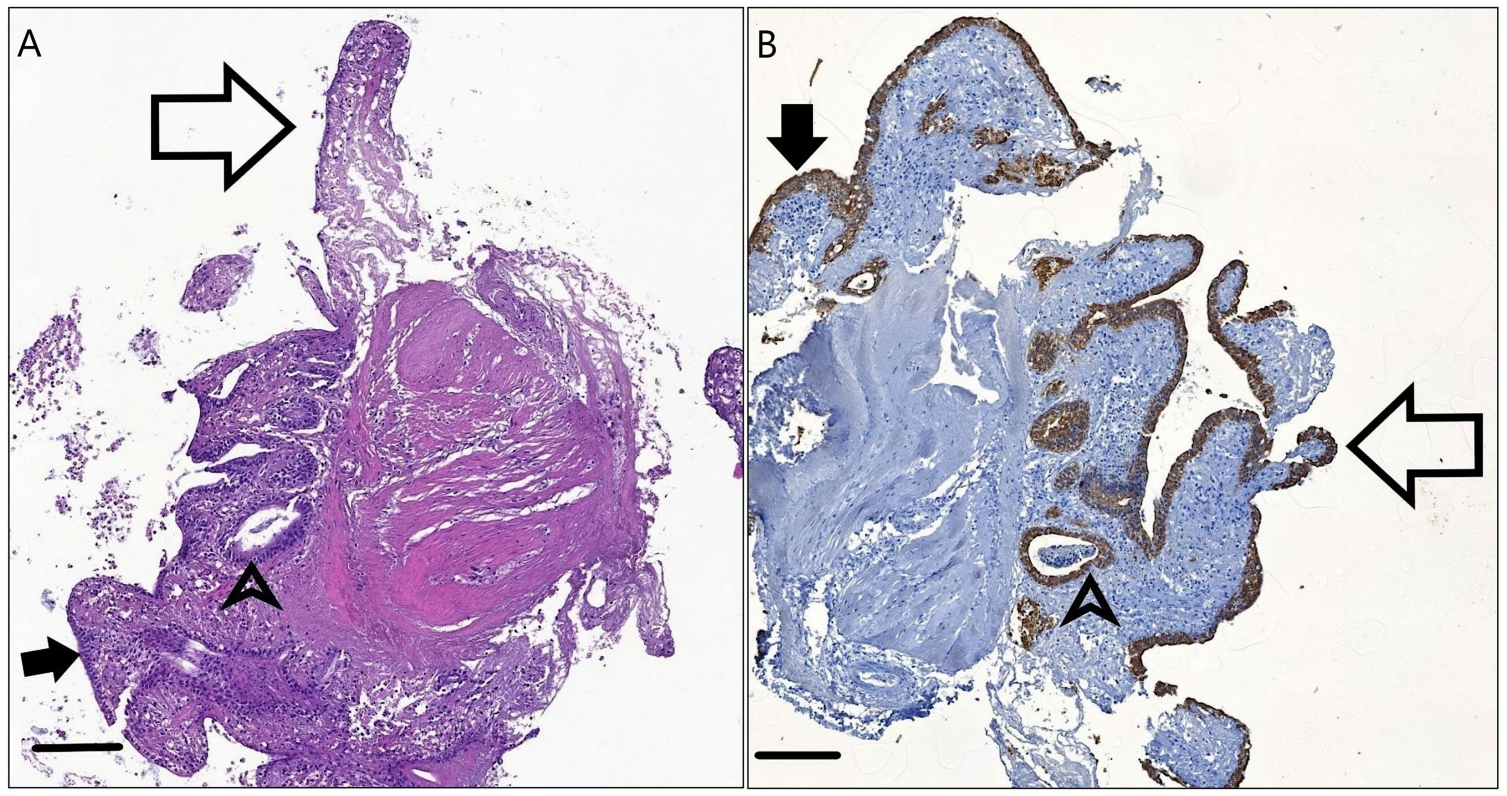
H&E stained **(A)** and Pan-cytokeratin stained **(B)** sections of the 1.5 mm explants after 12 h incubation. The physiologic columnar or cuboidal epithelial morphology (arrowheads) is visible on the surface (full arrows) and within the crypts (arrowheads). Remnants of the intestinal villi are present (empty arrows.) Bar line = 100 μm.

The 1.5 mm explants after 12 h incubation showed retained villous architecture to a certain degree and an epithelial lining, which was mostly columnar. Pan-cytokeratin staining highlighted presumably intact surface enterocytes, crypt epithelial cells and a few Goblet cells. A mild mononuclear infiltrate is visible in the lamina propria along with a small to moderate amount of tissue debris (Figure 4).

### 3.3 Study 3

**Figure 5:**
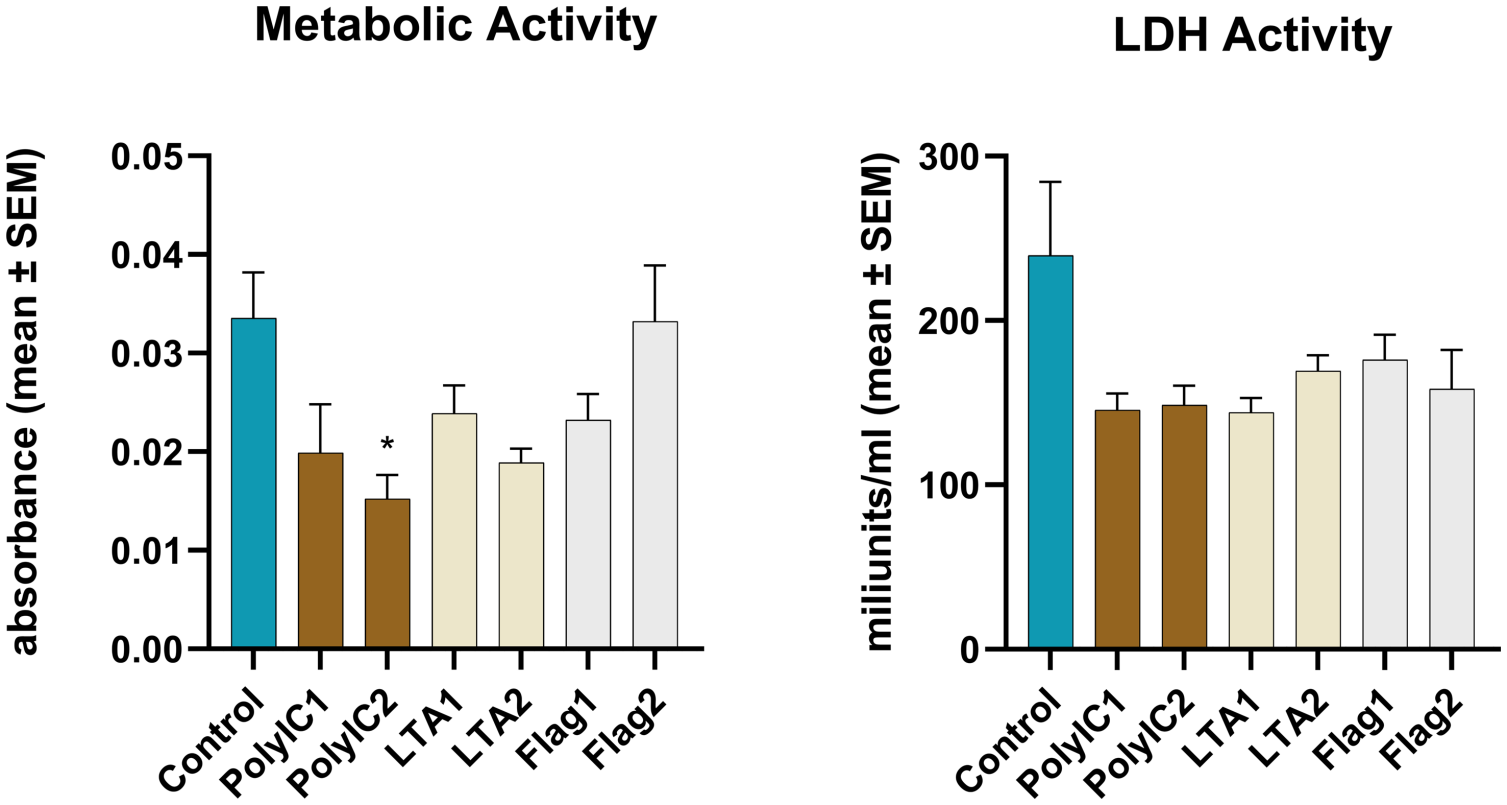
Metabolic activity measured by CCK-8 assay and extracellular lactate dehydrogenase (LDH) activity measured by an enzyme kinetic photometric assay of the 1.5 mm explants. PolyIC1 = 50 μg/mL polyinosinic-polycytidylic acid, PolyIC2 = 100 μg/mL polyinosinic-polycytidylic acid, LTA1 = 10 μg/mL *Staphylococcus aureus* lipoteichoic acid, LTA2 = 50 μg/mL *Staphylococcus aureus* lipoteichoic acid, Flag1 = 100 ng/mL *Salmonella Typhimurium* flagellin, Flag2 = 250 ng/mL *Salmonella Typhimurium* flagellin. Mean (*n* = 5/group) ± SEM.

The CCK-8 assay carried out immediately after 12 h PAMP (LTA, flagellin or poly I:C) exposures showed that the higher concentration of poly I:C treatment induced a significant decrease in the cellular metabolic activity compared to the control (*p* = 0.032) (Figure 5). No significant change was observed between PAMP-exposed and control explants in case of the lactate dehydrogenase activity (Figure 5).

**Figure 6:**
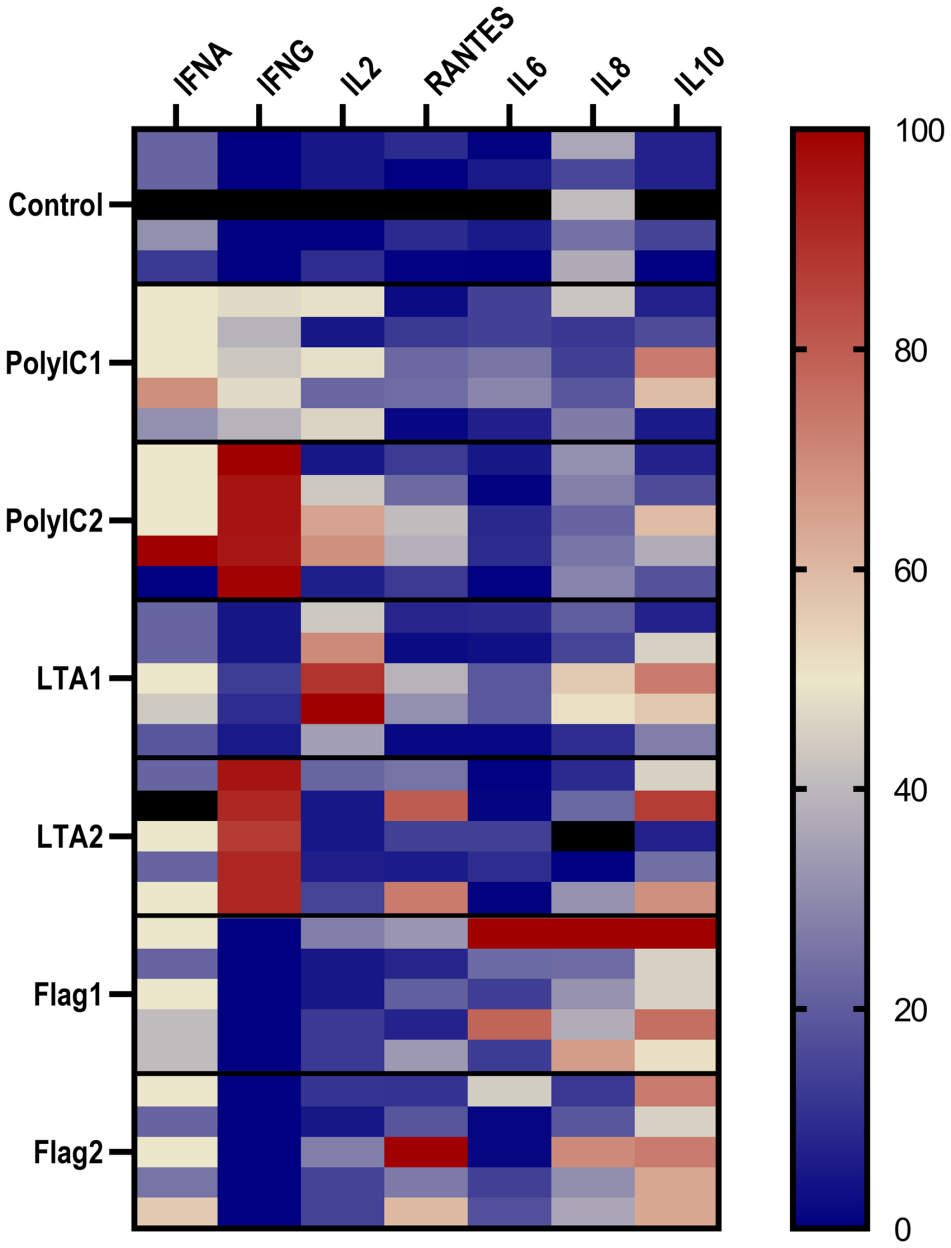
The heatmap represents the normalized relative percentages of the investigated cytokines. IFNA, interferon-*α*; IFNG, interferon-γ; RANTES, regulated on activation, normal T cell expressed and secreted; IL2, interleukin-2; IL6, interleukin-6; IL8, interleukin-8; IL10, interleukin-10; IL2, interleukin-2; PolyIC1 = 50 μg/mL polyinosinic-polycytidylic acid, PolyIC2 = 100 μg/mL polyinosinic-polycytidylic acid, LTA1 = 10 μg/mL *Staphylococcus aureus* lipoteichoic acid, LTA2 = 50 μg/mL *Staphylococcus aureus* lipoteichoic acid, Flag1 = 100 ng/mL *Salmonella Typhimurium* flagellin, Flag2 = 250 ng/mL *Salmonella Typhimurium* flagellin.

**Figure 7:**
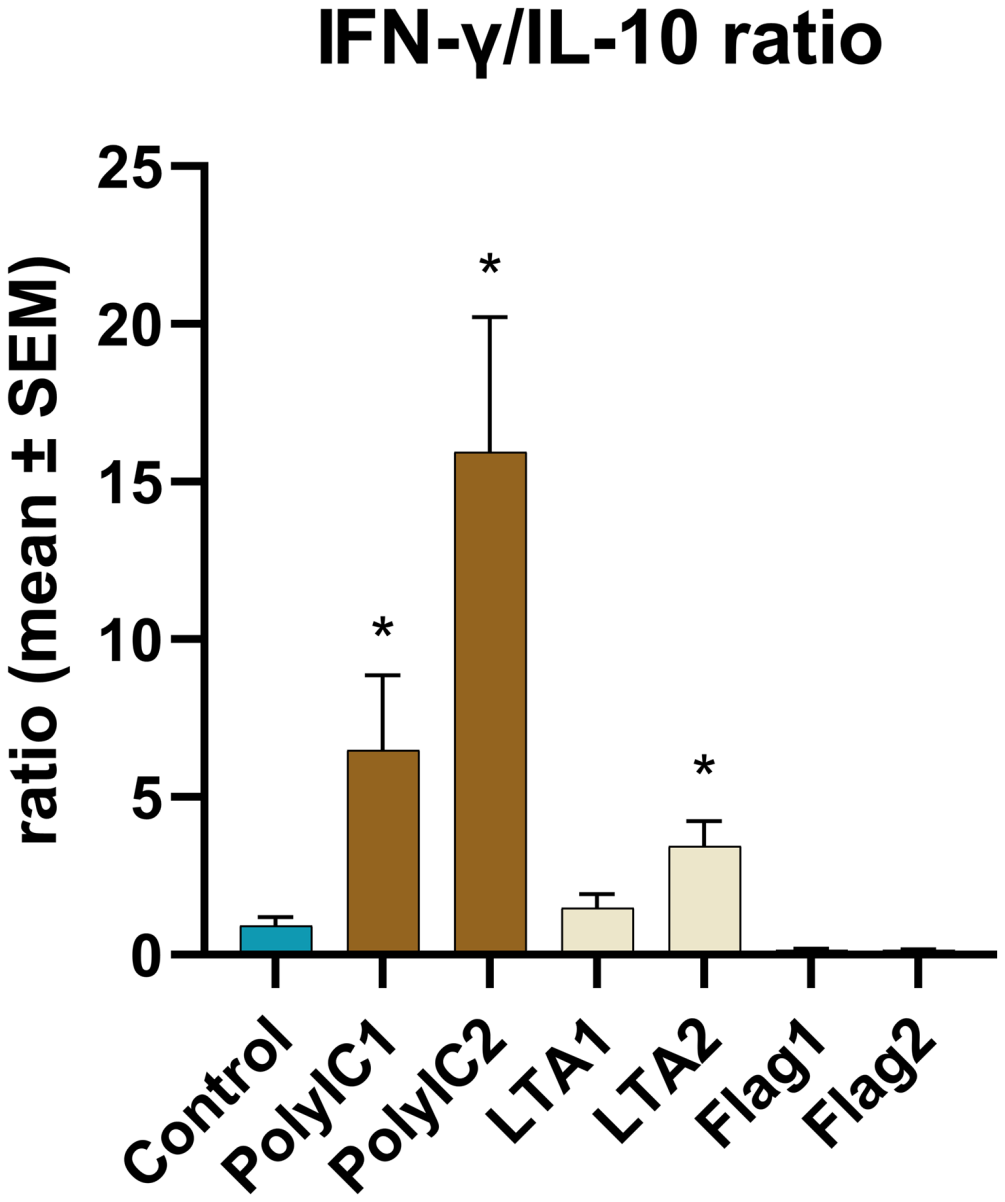
IFN-γ/IL-10 ratio. PolyIC1 = 50 μg/mL polyinosinic-polycytidylic acid, PolyIC 2 = 100 μg/mL polyinosinic-polycytidylic acid, LTA1 = 10 μg/mL *Staphylococcus aureus* lipoteichoic acid, LTA2 = 50 μg/mL *Staphylococcus aureus* lipoteichoic acid, Flag1 = 100 ng/mL *Salmonella Typhimurium* flagellin, Flag2 = 250 ng/mL *Salmonella Typhimurium* flagellin. Mean (*n* = 5/group) ± SEM, * *p* < 0.05.

IFN-*α* concentration was significantly increased by 50 μg/mL poly I:C treatment. LTA and poly I:C elevated IFN-*γ* at both applied concentrations. A significant increase was induced by LTA (10 μg/mL) for IL-2, whereas IL-6 was elevated by 100 ng/mL flagellin and 50 μg/mL poly I:C. In the case of flagellin treatment, IL-10 was up-regulated at both concentrations, while the higher concentration of poly I:C and flagellin increased the levels of RANTES (Figure 6, Table 1). The IFN-γ/IL-10 ratio was significantly increased by the higher applied concentration of LTA and in case of poly I:C in a concentration-dependent manner (Figure 7, Table 1). No significant change in the IL-8 concentration was induced by either of the applied treatments (Figure 6, Table 1).

**Table 1:** Changes with *p* values caused by the selected PAMPs compared to the control group.

Parameters	Supplements					
	Poly I:C (μg/mL)		Lipoteichoic acid (μg/mL)		Flagellin (ng/ml)	
	50	100	10	50	100	250
Metabolic activity		↓ 0.032				
IFN-α	↑ 0.024					
IFN-γ	↑ 0.014	↑ 0.015	↑ 0.015	↑ 0.013		
IL-2			↑ 0.019			
RANTES		↑ 0.016				↑ 0.016
IL-6	↑ 0.016				↑ 0.016	
IL-10					↑ 0.018	↑ 0.018
IFN-γ/IL-10 ratio	↑ 0.018	↑ 0.018		↑ 0.027		

Poly I:C, polyinosinic:polycytidylic acid.

## 4 Discussion

Explant systems comply with the R principles of laboratory animal experiments since they enable the preparation of multiple explants from a single animal while providing a tissue-specific response within a regulated environment (7, 9). Therefore, animal gut explant studies were done on a large scale to investigate the effects of mycotoxins and pathogens and to assess the impact of specific dietary components (10, 11, 16). The merit of the immersion technique is that the liquid phase, the intestinal fluid of the intestine is more accurately modeled in contrast to air-liquid interface cultures. Nevertheless, preventing anoxia-induced damage to the explants is challenging (7). Therefore, the authors aimed to follow the histology and viability parameters of 1 and 2 mm diameter sized smaller and bigger explants to describe possible size-dependent alterations. In view of the fact that 1 mm, smaller sized explants are hard to obtain and visualize during the change of the media the authors opted for the smallest possible 1.5 mm diameter explants in Study 2. In study 2 the morphology of 1.5 mm sized explants was examined after 12 h incubation. In study 3 according to the results of the preliminary studies the authors aimed to describe the effects of PAMPs on the 1.5 mm sized explant model after 12 h culturing.

In the preliminary part of the present work, Study 1, routine histology showed altered epithelial morphology and damaged lamina propria layer after 24 h of culturing in case of both 1 and 2 mm explants. Different-sized explants showed different extent of alteration in favor of 1 mm explants. The changes in epithelial shapes are difficult to interpret, but it is conceivable that the cells took on a flatter morphology, particularly in case of the 2 mm explants to cover the basement membrane due to the absence of the detached adjacent cells, or that they became flatter through another mechanism. The cells had supposedly undergone apoptosis and necrosis in the lamina propria of the specimens for both explants as inferred mainly from the nuclear alterations. As the degradation is much more visible deeper in the propria, the lack of nutrients or anoxia is suspected to be the reason. Although appreciable villus is absent either in the 24 h or the 0 h control slides, the 1 and 2 mm explants maintained their epithelial layer. Crypt cellular debris and the lack of differentiated Goblet cells are presumably associated with epithelial injury in the 2 mm samples. Randall et al. described that explants generally smaller than 3 mm are easy to lose during processing and challenging to be embedded in an appropriate orientation, which along with the possible damage caused by the punch could describe the loss of the villi in the present study (7).

Moreover, the Giemsa stained 1 mm cultures proved further the viability of the epithelium and the superficial layers after multiple days of maintenance. After 2–3 days of culturing the 2 mm explants were fragmented, which is in line with the prompt decrease in the metabolic activity and increase in the extracellular LDH activity in case of these samples. Although the original metabolic activity of the cells of 2 mm explant was higher, these changes suggest that the smaller 1 mm ileal explants are more reliable under extended (12 h or above) culturing. Nevertheless, prolonged incubation is controversial because of the histological alterations observed after 24 h in both explants. Under hypoxic conditions, the proinflammatory response related genes of human mature dendritic cells are over-expressed (20). The Hypoxia inducible factor (HIF)-nuclear factor signaling, and the inflammation related Nuclear factor kappa-light-chain-enhancer of activated B cells (NF-κB), which element is crucial in TLR signal transduction, are closely connected. Under anoxia the proinflammatory cytokine gene expression is increased, which might assist tissue recovery under physiologic conditions (21). Therefore, to prevent the influence of excessive anoxia on inflammatory response, the authors suggest a shorter incubation time and thus applied 12 h incubation in Study 2. After this amount of time, the peak translation of cytokine proteins could be assessed according to studies on different cell lines (22). Practically, the authors found the 1.5 mm and higher diameter biopsy punch more convenient to use as 1 mm explants might be stuck to the plunger or lost during the change of media. To shorten the time spent on the excision and to further decrease the difference between explants when a high amount of treatment groups and replicates are required, 1.5 mm explants could be a choice with minimal compromise. Therefore, in Studies 2 and 3, the smallest feasible 1.5 mm explants were examined. The length of the incubation was also limited to 12 h to assess the morphological status of untreated 1.5 mm explants and further in Study 3 to determine their acute inflammatory response under PAMP exposure.

Study 3 shows that TLR agonists unequivocally induce specific cytokine responses. A previous chicken ileal explant culture study of Zhang et al. demonstrates that explants remain viable for 2 h under ambient gas pressure conditions. Furthermore, according to the cited article, the culture served as a proper model to evaluate inflammatory response induced by lipopolysaccharide (LPS) stimuli as they observed that 20 μg/mL LPS could elevate the gene expression of IL-1β and IL-8. In contrast, 50 μg/mL could significantly increase the extracellular LDH activity, indicating cell injury (10). Most of the described TLRs have been characterized in heterophils, macrophages and in the intestinal samples of chicken (23). Therefore the authors aimed to assess the inflammatory response of ileal explants exposed to TLR agonist PAMPs for 12 h of culturing: poly I:C, LTA of *Staphylococcus aureus* and *Salmonella Typhimurium* derived flagellin. A human intestinal explant study underpinned that PAMPs have an essential role in gut inflammatory homeostasis which could be described accurately by IFN-*γ*/IL-10 ratio. This ratio is of high importance and indicative of LPS-dependent decrease in the constitutively expressed mucosal IL-10. This IL-10 decrease according to the authors could lead to IFN-γ elevation and concomitant damage and apoptosis of the human colon explant cells (24). The ratio similarly indicates inflammatory status in colitis models and an LPS-stimulated macrophage culture (25). In the present study flagellin induced significant elevation of IL-6, IL-10, and RANTES levels, although the IFN-γ/IL-10 was not altered. This observation might be related to the fact that TLR5, the Toll-like receptor of flagellin is confined to the basolateral side of enterocytes in human (26). The localization of this receptor has not been investigated, and this similarity has not been disproved in chickens so far. Therefore, flagellin might act on a more permeable epithelial barrier and stimulates a niche of cells deeper in the mucosa. Meanwhile, LTA and Poly I:C possibly induce TLR signals in enterocytes irrespective of the polarity. The capability of Poly I:C to induce inflammation on primary cell cultures of chicken origin was confirmed; however, its effect in mammalian intestinal *ex vivo* studies is controversial as it caused no cytokine production but increased the permeability of mucosal explants (3, 27, 28). LTA exposure has not been investigated before either on mammalian or on avian gut explant cultures, therefore the effect of LTA to increase the secreted IFN-*γ* and IL-2 concentrations and to elevate the IFN-γ/IL-10 ratio was first described in the present study. The elevation of the latter parameter might render LTA and also poly I:C challenge suitable for screening of anti-inflammatory candidates according to the mammalian model systems of Ma et al. In the experiment of the cited authors, IFN-γ/IL-10 ratio was used to quantify LPS or dysbiosis induced colonic and systemic pro-inflammatory response and concomitant neuroinflammation which were counteracted by probiotic bacterial strains (25).

The authors previously studied the effects of the listed PAMPs on chicken 2D and 3D primary hepatocyte—non-parenchymal cell co-cultures. In that experiment, the IL-8 production of 3D cultures was significantly decreased by the administered endotoxins, LPS and LTA. Meanwhile, only the higher LTA concentration was able to cause an increase in the IL-8 secretion of 2D cultures, and the treatment diminished the IL-6 level of 3D cultures. However, both 2 and 3D cultures responded under flagellin and poly I:C exposure with the elevation of IL-6 concentration and 50 μg/mL poly I:C raised even the IL-8 secretion of the 3D model developed with magnetic bioprinting (3). These primary cell culture studies further highlight that the polarity of defined cell types in a model highly determines the inflammatory response. The gut explants secrete cytokines and growth factors that are specific to the mucosal membrane and not to a particular cell type. This is an advantage of these models as several proinflammatoty interleukins are not or not constitutively secreted by single cell types e.g., human enterocytes or macrophages (29, 30).

In contrast, IFN-*γ* and IL-6 are secreted in small concentrations by the intestinal epithelial cells (29). Therefore, the effect of different agents and anti-inflammatory drugs is commonly tested also on intestinal *ex vivo* cultures to gain more representative data about the response of the complete gut mucosa (9). In the present investigation a marked significant increase in IFN-*γ* level was induced by LTA and poly I:C at both applied doses, while IFN-*α* concentration was considerably increased by 50 μg/mL poly I:C treatment. Concerning IL-2 concentration, 10 μg/mL LTA had an increasing effect, whereas 100 ng/mL flagellin and 50 μg/mL poly I:C elevated the level of IL-6. When flagellin was administered, IL-10 concentration was elevated at both doses, and RANTES levels rose in response to the higher poly I:C and flagellin concentrations. Elevated LTA and poly I:C concentrations considerably and concentration-dependently raised the IFN-γ/IL-10 ratio compared to the control. Therefore, the application of the examined agents, LTA, poly I:C and flagellin could induce a satisfactory proinflammatory response on chicken ileal explant cultures based on the cytokines of interest.

The applied 1 and 2 mm explants showed a mild alteration of the epithelium and notable damage of the lamina propria after 24 h of culturing, meanwhile after 12 h culturing in Study 2 the 1.5 mm explants possessed a mostly columnal epithelial lining with moderate impairment in the propria. Mucosal damage is a characteristic of most of the gut leakage models as it captures the two-hit nature of gut inflammation. This aspect of inflammatory bowel disease is exploited and emphasized in case of the mouse model of dextran sodium sulfate (DSS) induced ulcerative colitis. DSS primarily induces damage of the epithelial barrier and therefore facilitates the intestinal pathogens and their antigens to reach the deeper layers of the mucosa (31). At the site of the inflammation, damage associated molecular patterns (DAMPs) are released from the destructed cells. These conjointly with PAMPs are triggers in regulating inflammation and thus play a substantial role in gastrointestinal diseases, e.g., inflammatory bowel disease. The faulty healing of the gut barrier plays an unequivocal role in maintaining inflammatory bowel disease (32). This tissue damage is indeed a characteristic of the present *in vitro* model, which is, from one hand, a limitation of it. However, the involvement of DAMPs and impaired tissue integrity related factors could make the explant model even more reasonable if mild damage of the propria and the epithelial layer are not objections for the research setting. Especially if the study aims to investigate the anti-inflammatory potential of drugs and drug metabolites that could not penetrate through the intact mucosal barrier (e.g., host defense peptides) (33).

## 5 Conclusion

The present data underpins that biopsy punches genuinely used for the section of gut explants could be applied to obtain tissue samples with identical size and similar morphology. Chicken gut explants greater in size showed damage sooner during culturing according to the macroscopic, microscopic morphology and the cell viability assays. 1–1.5 mm diameter explants are comparable in size to spheroid cultures, and according to H&E and pan-cytokeratin staining, they better retain their histomorphology, making them a suitable model for 12 h of culturing. Under these conditions PAMPs induced remarkable changes compared to the control group in the measured cytokines. At least one concentration of poly I:C elevated the IFN-*α*, IFN-*γ*, RANTES and IL-6 level. Furthermore, LTA increased the concentration of IFN-γ and IL-2, while RANTES, IL-6 and IL-10 elevation was noted in the case of flagellin exposure. Moreover, poly I:C and LTA increased the IFN-γ/IL-10 ratio indicative of the intestinal inflammatory status. It substantiates that the application of LTA, poly I:C and flagellin on chicken ileal explant culture renders an appropriate model to assess the effects of the future nutraceuticals and drugs of the poultry industry with possible anti-inflammatory effect. Furthermore, the authors suggest similar simplistic models with low technical requirements to be applied for modeling enteritis of mammalian species.

## Data Availability

The raw data supporting the conclusions of this article will be made available by the authors, without undue reservation.

## References

[1] Takahashi I Kiyono H. Gut as the largest immunologic tissue. JPEN J Parenter Enteral Nutr. (1999) 23:S7–S12. doi: 10.1177/014860719902300503, PMID: 10483885

[2] Mowat AM. Anatomical basis of tolerance and immunity to intestinal antigens. Nat Rev Immunol. (2003) 3:331–41. doi: 10.1038/nri1057, PMID: 12669023

[3] Sebők C Tráj P Vörösházi J Mackei M Papp M Gálfi P. Two sides to every question: attempts to activate chicken innate immunity in 2D and 3D hepatic cell cultures. Cells. (2021) 10:1910. doi: 10.3390/cells10081910, PMID: 34440679 PMC8394239

[4] Wickramasuriya SS Park I Lee K Lee Y Kim WH Nam H. Role of physiology, immunity, microbiota, and infectious diseases in the gut health of poultry. Vaccine. (2022) 10:172. doi: 10.3390/vaccines10020172, PMID: 35214631 PMC8875638

[5] Abreu MT. Immunologic regulation of toll-like receptors in gut epithelium. Curr Opin Gastroenterol. (2003) 19:559–64. doi: 10.1097/00001574-200311000-00008, PMID: 15703605

[6] Hickey JW Becker WR Nevins SA Horning A Perez AE Zhu C. Organization of the human intestine at single-cell resolution. Nature. (2023) 619:572–84. doi: 10.1038/s41586-023-05915-x, PMID: 37468586 PMC10356619

[7] Randall KJ Turton J Foster JR. Explant culture of gastrointestinal tissue: a review of methods and applications. Cell Biol Toxicol. (2011) 27:267–84. doi: 10.1007/s10565-011-9187-5, PMID: 21384137

[8] Liu C Yang J. Enteric glial cells in immunological disorders of the gut. Front Cell Neurosci. (2022) 16:895871. doi: 10.3389/fncel.2022.895871, PMID: 35573829 PMC9095930

[9] Russo I Zeppa P Iovino P Del Giorno C Zingone F Bucci C. The culture of gut explants: a model to study the mucosal response. J Immunol Methods. (2016) 438:1–10. doi: 10.1016/j.jim.2016.07.004, PMID: 27475701

[10] Zhang Q Eicher SD Ajuwon KM Applegate TJ. Development of a chicken ileal explant culture model for measurement of gut inflammation induced by lipopolysaccharide. Poult Sci. (2017) 96:3096–103. doi: 10.3382/ps/pex160, PMID: 28633471

[11] Costa MO Harding JCS Hill JE. Development and evaluation of a porcine in vitro colon organ culture technique. In Vitro Cell Dev Biol Anim. (2016) 52:942–52. doi: 10.1007/s11626-016-0060-y 27338737

[12] Neckel PH Just L. Organotypical tissue cultures from fetal and neonatal murine Colon. Methods Mol Biol. (2016) 1422:41–7. doi: 10.1007/978-1-4939-3603-8_5, PMID: 27246021

[13] Bareiss PM Metzger M Sohn K Rupp S Frick JS Autenrieth IB. Organotypical tissue cultures from adult murine colon as an in vitro model of intestinal mucosa. Histochem Cell Biol. (2008) 129:795–804. doi: 10.1007/s00418-008-0405-z, PMID: 18320204 PMC2584443

[14] Mackei M Molnár A Nagy S Pál L Kővágó C Gálfi P. Effects of acute heat stress on a newly established chicken hepatocyte—nonparenchymal cell co-culture model. Animals. (2020) 10:409. doi: 10.3390/ani10030409, PMID: 32121577 PMC7142495

[15] Udden SMN Waliullah S Harris M Zaki H. The ex vivo Colon organ culture and its use in antimicrobial host defense studies. J Vis Exp. (2017) 120:55347. doi: 10.3791/55347-v, PMID: 28287576 PMC5408962

[16] Peñaranda DS Bäuerl C Tomás-Vidal A Jover-Cerdá M Estruch G Pérez Martínez G. Intestinal explant cultures from gilthead seabream (*Sparus aurata*, L.) allowed the determination of mucosal sensitivity to bacterial pathogens and the impact of a plant protein diet. Int J Mol Sci. (2020) 21:7584. doi: 10.3390/ijms21207584, PMID: 33066515 PMC7588912

[17] Lim JH Davis GE Rue TC Storm DR. Human sinonasal explant system for testing cytotoxicity of intranasal agents. Int Forum Allergy Rhinol. (2012) 2:63–8. doi: 10.1002/alr.20110, PMID: 22170775 PMC3274569

[18] Kolf-Clauw M Castellote J Joly B Bourges-Abella N Raymond-Letron I Pinton P. Development of a pig jejunal explant culture for studying the gastrointestinal toxicity of the mycotoxin deoxynivalenol: histopathological analysis. Toxicol In Vitro. (2009) 23:1580–4. doi: 10.1016/j.tiv.2009.07.015, PMID: 19607908

[19] R Core Team. R: A language and environment for statistical computing. Vienna, Austria: R Foundation for Statistical Computing (2020).

[20] Blengio F Raggi F Pierobon D Cappello P Eva A Giovarelli M. The hypoxic environment reprograms the cytokine/chemokine expression profile of human mature dendritic cells. Immunobiology. (2013) 218:76–89. doi: 10.1016/j.imbio.2012.02.002, PMID: 22465745

[21] Pham K Parikh K Heinrich EC. Hypoxia and inflammation: insights from high-altitude physiology. Front Physiol. (2021) 12:676782. doi: 10.3389/fphys.2021.676782, PMID: 34122145 PMC8188852

[22] Israelsson P Dehlin E Nagaev I Lundin E Ottander U Mincheva-Nilsson L. Cytokine mRNA and protein expression by cell cultures of epithelial ovarian cancer—methodological considerations on the choice of analytical method for cytokine analyses. Am J Reprod Immunol. (2020) 84:e13249. doi: 10.1111/aji.13249, PMID: 32307767

[23] Kannaki TR Reddy MR Shanmugam M Verma PC Sharma RP. Chicken toll-like receptors and their role in immunity. Worlds Poult Sci J. (2010) 66:727–38. doi: 10.1017/S0043933910000693

[24] Jarry A Bossard C Bou-Hanna C Masson D Espaze E Denis MG. Mucosal IL-10 and TGF-beta play crucial roles in preventing LPS-driven, IFN-gamma-mediated epithelial damage in human colon explants. J Clin Invest. (2008) 118:1132–42. doi: 10.1172/JCI32140, PMID: 18259614 PMC2230656

[25] Ma X Shin YJ Jang HM Joo MK Yoo JW Kim DH. Lactobacillus rhamnosus and *Bifidobacterium longum* alleviate colitis and cognitive impairment in mice by regulating IFN-γ to IL-10 and TNF-α to IL-10 expression ratios. Sci Rep. (2021) 11:20659. doi: 10.1038/s41598-021-00096-x, PMID: 34667205 PMC8526673

[26] Hug H Mohajeri MH La Fata G. Toll-like receptors: regulators of the immune response in the human gut. Nutrients. (2018) 10:203. doi: 10.3390/nu10020203, PMID: 29438282 PMC5852779

[27] Brand RM Biswas N Siegel A Myerski A Engstrom J Jeffrey Metter E. Immunological responsiveness of intestinal tissue explants and mucosal mononuclear cells to ex vivo stimulation. J Immunol Methods. (2018) 463:39–46. doi: 10.1016/j.jim.2018.08.009, PMID: 30218652

[28] Moyano-Porcile V Olavarría-Ramírez L González-Arancibia C Bravo JA Julio-Pieper M. Short-term effects of poly(I:C) on gut permeability. Pharmacol Res. (2015) 101:130–6. doi: 10.1016/j.phrs.2015.06.016, PMID: 26145280

[29] Noel G Baetz NW Staab JF Donowitz M Kovbasnjuk O Pasetti MF. A primary human macrophage-enteroid co-culture model to investigate mucosal gut physiology and host-pathogen interactions. Sci Rep. (2017) 7:45270. doi: 10.1038/srep45270, PMID: 28345602 PMC5366908

[30] Daig R Rogler G Aschenbrenner E Vogl D Falk W Gross V. Human intestinal epithelial cells secrete interleukin-1 receptor antagonist and interleukin-8 but not interleukin-1 or interleukin-6. Gut. (2000) 46:350–8. doi: 10.1136/gut.46.3.350, PMID: 10673296 PMC1727863

[31] Chassaing B Aitken JD Malleshappa M Vijay-Kumar M. Dextran sulfate sodium (DSS)-induced colitis in mice. Curr Protoc Immunol. (2014) 104:15.25.1–15.25.14. doi: 10.1002/0471142735.im1525s104, PMID: 24510619 PMC3980572

[32] Boyapati RK Rossi AG Satsangi J Ho GT. Gut mucosal DAMPs in IBD: from mechanisms to therapeutic implications. Mucosal Immunol. (2016) 9:567–82. doi: 10.1038/mi.2016.14, PMID: 26931062

[33] Mátis G Tráj P Hanyecz V Mackei M Márton RA Vörösházi J. Immunomodulatory properties of chicken cathelicidin-2 investigated on an ileal explant culture. Vet Res Commun. (2024) 48:2527–35. doi: 10.1007/s11259-024-10428-7, PMID: 38871866 PMC11315780

[34] Mátis G Sebők Cs Horváth DG Márton RA Mackei M Vörösházi J. Miniature chicken ileal explant culture to investigate the inflammatory response induced by pathogen associated molecular patterns. [Preprint] (2024). Available at: https://ssrn.com/abstract=4685306 10.3389/fvets.2025.1484333 PMC11960747 40171408

